# Queer in Chem: Q&A with Professor Abhik Ghosh

**DOI:** 10.1038/s42004-023-00966-7

**Published:** 2023-09-30

## Abstract

*Abhik Ghosh* grew up in Kolkata, India, and is a Professor of inorganic and materials chemistry at UiT—The Arctic University of Norway. His research interests lie at the intersection of inorganic, materials and computational chemistry.

**Figure:**
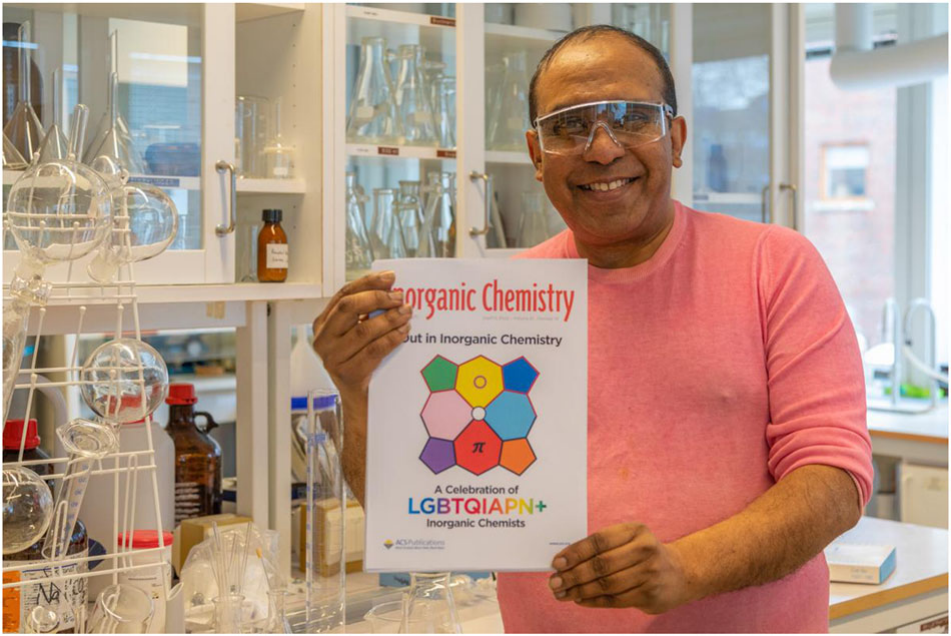
Stina Gulbrandsen, UiT The Arctic University of Norway]

Abhik was one of the first to apply modern ab initio and density functional theory methods to bioinorganic chemistry, especially hemes and other metalloporphyrins^1^. In the 1990s, his calculations on the potential energy surface of carbonmonoxyheme facilitated the abandonment of the steric model for diatomic ligand discrimination by heme proteins^2^. Instead, hydrogen bonds in the distal pocket were shown to be primarily responsible for the selective binding of O_2_ over CO and NO. The calculations provided a much-needed theoretical underpinning as the field of heme-based sensors came of age in that period^3^. Abhik is one of today’s leading thinkers on so-called noninnocent ligands, which do not allow an unambiguous assignment of the oxidation state of a coordinated metal. He developed a set of criteria that help inorganic chemists to classify a transition metal system as innocent or otherwise^4^. He has also used advanced ab initio calculations to quantify the phenomenon, ranking a set of metallocorroles according to their degree of noninnocence^5^. In recent years, Abhik has pioneered the field of 5d metallocorroles, which exhibit promise as new building blocks for cancer phototherapeutics^6^. In 2022, he won the Hans Fischer Career Award for Lifetime Achievements in Porphyrin Chemistry. Abhik takes a certain pride in being a fluent speaker of Sanskrit, and not long ago, he and Stanford University linguist Paul Kiparsky made waves with their reflections on the possible role of Sanskrit as a source of inspiration behind Mendeleev’s periodic table^7^.

Why did you choose to be a scientist?

I can’t point to a single factor or event that dictated my career choice. To some extent, I followed in my dad’s footsteps. My dad was a geologist, and my mom and I sometimes joined him on his field trips. These instilled in me a love of the natural world—plants, animals, rocks, everything. Even today, birding is my major hobby outside science. That said, I almost did not become a scientist. Most so-called good students in India go for professional fields such as engineering and medicine—at least they did in the 1980s when I finished high school. I resisted that pressure—it took some courage. Aside from that, I had many superb teachers all through my career. One of them was the late Harvard-trained physical organic chemist Amareshwar Chatterjee at Jadavpur University. Another was the photochemist Krishna Kamini Rohatgi-Mukherjee. Their love of science, I suppose, rubbed off on me.

What scientific developments are you most excited about?

There’s so much great stuff happening … I find the field of metals in medicine to be fascinating, especially as it relates to developing new therapeutic and diagnostic agents for cancer. There’s the coordination chemistry part. Then there are challenges involving bioconjugation and nanoconjugation. You can think of strategies to target the tumor microenvironment, or multiple ways of attacking a tumor—photodynamic and photothermal therapies, radiosensitization, etc. The possibilities are endless.

All that remains a dream for me now. So far, my achievements are primarily in the area of coordination chemistry. Our 5d metallocorroles are rather curious constructs in which a sterically constrained corrole encapsulates a large 5d transition metal. Despite the steric misfit, the complexes by and large are exceedingly rugged and, what’s more, effective triplet photosensitizers. My dream is to develop considerably more sophisticated analogs of these complexes for multimodal cancer therapy and diagnostics (theranostics).

What directions do you think your research should go in?

The metals in medicine direction is obviously very attractive for me. However, I work at a small university with somewhat limited facilities and modest funding, so a highly competitive area doesn’t seem like the best choice. Others at larger, richer universities will easily outdistance me.

Basic research in coordination chemistry, supplemented by synchrotron-based analyses and theory, therefore, will probably be a mainstay for me. Metals in medicine will probably be more of an inspiration rather than an end goal for me. In other words, potential applications in medicine will provide the motivation for fundamental discoveries in coordination chemistry.

How does your queer or trans identity intersect with your identity as a scientist?

The idea that one’s personal and professional lives need to sit together in a harmonious manner is a no-brainer. In Norway, it’s rather simple. Friends, colleagues, and students know I’m queer—bi but mostly gay—and it’s pretty much a nonissue. It feels a bit odd, therefore, to give this interview, to stand out as a queer icon, as it were.

Globally, however, the overwhelming majority of queer people remain closeted, and I feel I must do my part to give a voice to this vast group that remains voiceless^8^. On a happier note, LGBTQ+ people in my own country, India, have won increasing acceptance in recent years. According to a recent Pew Global Survey, 53% of Indians now support same-sex marriage! Even so, many queer Indian scientists still conceal their sexuality in professional settings. That needs to change. The message needs to be loud and clear: we’re here, we’ve always been here, and we’re as good as anyone else.

Homophobia does persist in Norway (and other Western countries) in certain immigrant communities. I count a number of people in these communities as friends. Upon reading this interview, they will probably see me in a somewhat different light, but hopefully still as their loyal friend and supporter.

Have you faced any challenging situations in your professional life as a result of your queer or trans identity? What did you or others learn from these experiences?

No, nothing major as a result of being queer. The worst that I have experienced is that a handful of collaborators have “ghosted” me upon learning that I’m queer! It’s probably just as well!

I have, however, faced an *unusual* degree of obstruction, bullying, and harassment from a small number of "colleagues"—using the word in a broad sense and without reference to a specific place or time. The incidents have taken a devastating toll on my career and on my mental and physical well-being. As a result, I have, at multiple junctures, considered leaving science. As far as I can tell, the incidents were driven by petty jealousies and resentment, along with a dash of racism, rather than homophobia, which brings me to an important point: it seems to me that here in the most liberal corner of the West, racism is a bigger problem than homophobia. A major part of the problem is that racism is a taboo subject in many European countries: "Racism in Norway? You can’t be serious!" The reality is somewhat different^9,10^.

On the positive side, I enjoy many, many wonderful friendships and collaborative relationships with scientists from all over the world. Some of them have lasted decades and will hopefully continue for the rest of our lives. My interactions with students at UiT—and I have taught a generation by now—have also been exceedingly positive. Many ended up conducting research projects in my laboratory. It’s been a privilege to share my love of science and the thrill of discovery with them. Without that camaraderie, I probably would not have survived the harassment that has otherwise darkened my professional life.

Lessons learned? Do not suffer alone and in silence. Find out who your allies are and confide in them. Seek early intervention from authorities—I realize that’s easier said than done, but it’s very important!

How can individual scientists support and celebrate their LGBTQ+ colleagues? How do you lift yourself and potentially the LGBTQ+ community up to thrive in chemical research?

There are so many ways: You can highlight contributions by queer scientists in classes and scientific conferences. My favorite example is the late Martin Gouterman—a foundational figure in porphyrin chemistry and spectroscopy and one of the first openly gay chemists^11^. I make it a point to mention that there’s also an entire generation of individuals whom I cannot acknowledge by name (for privacy reasons), who contributed profoundly to science and yet died in loneliness and shame of HIV-AIDS.

Many academic institutions (and larger private companies) organize queer-themed seminars and social events as a means of promoting diversity and inclusion. Academic journals increasingly do the same with queer-themed virtual issues and special issues. Some are critical of the latter practice as an unwarranted intrusion of politics into science—it’s not a view that I share. Science does not take place in a vacuum: there’s nothing wrong with devoting a few pages to shine a light on the marginalized among us, much as we might to discuss, say, geopolitical developments that have a bearing on science. I am particularly proud of a Virtual Issue of *Inorganic Chemistry* titled "Out in Inorganic Chemistry: A Celebration of LGBTQIAPN+ Inorganic Chemists" that I recently edited with Bill Tolman^12^. In it, we highlighted contributions by some 37 queer chemists. In the 12 months since publication, our editorial was downloaded over 12,000 times! The substantial amount of feedback that we have received indicates that both our Editorial and the Virtual Issue as a whole have inspired legions of queer chemists and students around the world—especially in parts of the world where they remain oppressed and invisible. For us, it’s been a remarkable demonstration of the role that scientific journals can play in advancing diversity and inclusion.

Of course, not everyone has to go out of their way to support queer causes. Simple, verbal expressions of support for LGBTQ+ colleagues can go a long way toward creating a welcoming atmosphere.

What can employers do to make a difference for LGBTQ+ scientists?

That depends a lot on the nature and location of the organization, obviously. Regardless, employers can do a lot. The first order of business is to normalize queer employees: some people are queer, get over it! Setting up safe spaces for queer employees is a logical next step. Queer-themed lectures and social events are another possibility. In certain parts of the world, employers can and should take the lead in helping queer employees and students access basic sexual health care in the form of, say, condoms and PrEP (pre-exposure prophylaxis against HIV-AIDS). Finally, employers need to implement robust mechanisms for tackling and rooting out discrimination and harassment, especially as they relate to gender identity and sexuality.

*This interview was conducted by the editors of Communications Chemistry*.
